# Expressive suppression mediates the relationship between sleep quality and generalized anxiety symptomology

**DOI:** 10.1038/s41598-024-63939-3

**Published:** 2024-06-12

**Authors:** Robert C. A. Bendall, Sophie N. Elton, Alun T. L. Hughes

**Affiliations:** 1 Directorate of Psychology and Sport, School of Health and Society, University of Salford, Salford, UK; 2 Centre for Applied Health Research, University of Salford, Salford, UK; 3 School of Biological and Environmental Sciences, Liverpool John Moores University, Liverpool, UK; 4 Institute of Health Research, Liverpool John Moores University, Liverpool, UK

**Keywords:** Emotion regulation, Sleep quality, Anxiety, Expressive suppression, Cognitive reappraisal, Generalized anxiety disorder, Psychology, Human behaviour

## Abstract

Anxiety disorders are the most prevalent worldwide mental health disorder, resulting in high societal costs. Emotion regulation and sleep quality are associated with the development of psychopathologies including anxiety. However, it is unknown whether habitual emotion regulation strategy use can mediate the influence of sleep quality on anxiety symptomology. An opportunity sample in a healthy population completed the Pittsburgh Sleep Quality Index to provide a measure of sleep quality, the Emotion Regulation Questionnaire to assess habitual use of emotion regulation strategies, and the Generalized Anxiety Disorder Scale to record anxiety symptomology. Data were analysed using correlation and regression-based mediation analyses. Improved sleep quality was predictive of reduced habitual use of expressive suppression and reduced anxiety symptomology. Additionally, increased use of expressive suppression was predictive of greater anxiety symptomology. Cognitive reappraisal was not associated with sleep quality or anxiety severity. Further, novel findings using mediation analyses show that expressive suppression partially mediated the relationship between sleep quality and anxiety. Whilst longitudinal and experimental research are needed to establish causality, these findings suggest that simultaneously targeting improvements in sleep quality and the use of specific emotion regulation strategies, including expressive suppression, may improve the efficacy of interventions focussed on reducing anxiety-related symptomology.

## Introduction

Anxiety disorders are the most prevalent worldwide mental health disorder^1^. The absolute number of anxiety disorders increased by 50% between 1990 and 2019 demonstrating a vast societal burden^2,3^ as well as high financial costs to individuals^4^. Generalized anxiety disorder (GAD) is characterised by excessive worry and is associated with feelings of restlessness, fatigue, difficulty concentrating, irritability, muscle tension, and sleep disturbances^5^. Given that GAD has an onset typically evident in childhood or adolescence^6^, has high comorbidity with other mental health disorders^7^, and has been shown to precede the onset of other psychiatric disorders^7^, research investigating the aetiology of GAD has potential to aid in the development of earlier and more efficacious interventions and treatments for a wide range of mental health disorders.

Sleep is crucial for physical and psychological health^8^. People with anxiety frequently exhibit sleep disturbances including insomnia and poor sleep quality^9^. For instance, longitudinal research has investigated the relationship between anxiety and sleep problems. Whilst three quarters of all primary care patients (74%) reported sleep problems, participants with a diagnosis of either GAD or Post-Traumatic Stress Disorder were twice as likely to have sleep problems than those without^10^. Anxiety symptomology has also been shown to be exacerbated when people report a greater number of insomnia-related symptoms^11^. Subclinical anxiety symptomology has also been associated with sleep. For instance, higher levels of state anxiety have been shown to be associated with worse sleep quality in community volunteers^12,13^, healthy older adults^14^, and university students^15^. Moreover, it has been argued that the relationship between insomnia and affective disorders is bidirectional, suggesting that anxiety and depression predict the prevalence of insomnia, and that insomnia can also predict anxiety symptomology^16^. Indeed, anxiety has been shown to be instigated or exacerbated by sleep deprivation^17,18^, whilst meta-analytic work has shown that acute sleep deprivation predicts state anxiety^19^. Similarly, people with insomnia and sleep disturbances often excessively worry about their sleep requirements and the consequences if they are not met^20^. This excessive worry tends to increase arousal and emotional distress, therefore further adding to an individual’s inability to fall asleep and maintaining anxiety.

Difficulties with emotion regulation (ER) are a common feature of anxiety^21^, and theoretical models propose that ER functions in both the development and maintenance of anxiety disorders^22^. ER is concerned with how and when people control their emotions, with different strategies employed to manage one’s feelings. Two strategies that have received the most research focus are cognitive reappraisal and expressive suppression. Cognitive reappraisal is defined as a cognitive-linguistic strategy used to change the meaning of a situation and emotional response^23^. Gross and John^24^ suggest that repeated use of cognitive reappraisal can have positive effects such as improved control of emotion, interpersonal functioning, and psychological and physical health. Expressive suppression is characterised by the inhibition of responses such as facial expressions and gestures which are related to emotional expression^23^. It is suggested that long-term use of this technique has negative effects such as reduced control over one’s emotions, lower interpersonal functioning, and increased rates of depression^24^. Habitual use of different ER strategies has been shown to either act as a risk factor for the development of affective disorders, including anxiety, or as to act as a protective factor^21^. For instance, increased use of expressive suppression, often cited as a maladaptive ER strategy, is associated with higher levels of internalizing symptoms, including anxiety. In contrast, people who more frequently use cognitive reappraisal, which is an adaptive ER strategy, exhibit fewer symptoms^21,25–29^.

The associations between specific ER strategies and sleep quality have previously been investigated. For instance, increased habitual use of expressive suppression has been associated with reduced sleep quality, whereas habitual use of cognitive reappraisal is not related to sleep quality^28,30^. However, experimentally determined cognitive appraisal ability does correlate with self-reported sleep quality^31^. Intriguingly, an evening chronotype, which is frequently associated with reduced sleep quality^32–35^, is associated with increased use of expressive suppression, whilst a morning chronotype is associated with more frequent use of cognitive reappraisal^36,37^. Research investigating the associations between sleep quality and ER in people with anxiety symptomology is limited. Compared to a control group, people with GAD score higher on several sleep outcomes, demonstrating poorer sleep, but importantly, difficulties in ER were shown to mediate the relationship between GAD and some sleep outcomes (e.g., sleep disturbances, excess daytime sleepiness, daytime disfunction and perceived need for more sleep)^38^. However, this was not the case for all sleep outcomes, including subjective sleep quality, where difficulties in ER did not mediate the relationship with GAD. Crucially, this study did not specifically test different ER strategies, instead using a more general measure of ER difficulty. Therefore, whether different ER strategies mediate the relationship between sleep quality and GAD symptomology remains unknown.

One study that has focussed on different ER strategies investigated the associations between sleep, ER and affective disorders in adolescents^39^. Here it was shown that people with more severe sleep problems were more likely to meet the criteria for diagnosis of a mood or anxiety disorder whilst also reporting poorer ER strategy use. Explicitly, these people were less likely to use problem-solving and more likely to exhibit avoidance, suppression, rumination and acceptance. Moreover, sleep disturbances were shown to be associated with anxiety symptomology via greater use of suppression and rumination. These findings in adolescents suggest that specific ER strategies may be more difficult for adolescents with sleep deficits. Additional work is required to build upon these initial findings in adolescent populations to investigate which ER strategies mediate the associations between sleep quality and anxiety symptomology in healthy and clinical adult populations.

The precise nature of the relationship between sleep quality and anxiety symptomology, and whether or how this is mediated by specific ER strategies, remains inconclusive. Such research is important as it permits researchers to identify those people who are most vulnerable and therefore develop more targeted and personalised interventions specifically for those who demonstrate deficits in the use of specific ER strategies. Research in healthy populations may aid the development of future investigations regarding how these processes are altered in clinical populations, enhancing our understanding of the associations between sleep, ER and GAD and developing more efficacious interventions. The aim of the current study is to test the associations between sleep quality, habitual ER strategy use and anxiety symptomology in people without a diagnosed psychiatric condition or sleep disorder. It was predicted that improved sleep quality would be associated with reduced anxiety symptomology. Moreover, we predicted that increased habitual use of expressive suppression, most frequently a maladaptive ER strategy, would predict increased anxiety symptomology. In contrast, we predicted that increased habitual use of cognitive reappraisal, an adaptive ER strategy, would predict reduced anxiety symptomology. Currently it is not known whether habitual use of ER strategies can mediate the influence of sleep quality on anxiety symptoms, and we therefore extend this approach to test whether ER can mediate the influence of sleep quality on anxiety symptomology.

## Results

Descriptive statistics for the variables assessed in this study are presented in Table 1. All variables were assessed for normality using the Shapiro–Wilk test, and inspection of frequency histograms and Q-Q plots. These assessments indicated a non-normal distribution for all four variables and therefore Spearman’s Rank Order Correlation was used to investigate the relationships between them.

**Table 1:** Descriptive statistics for anxiety, emotion regulation and sleep quality variables.

Variable	Mean ± SEM	Shapiro–Wilk
GAD-7	5.7 ± 0.3	*W*(203) = 0.916, *p* < 0.0001
ERQ-CR	28.3 ± 0.5	*W*(203) = 0.976, *p* = 0.0016
ERQ-ES	14.2 ± 0.4	*W*(203) = 0.976, *p* = 0.0015
PSQI	7.1 ± 0.2	*W*(203) = 0.976, *p* = 0.0016

*GAD-7* Generalised Anxiety Disorder Questionnaire, *ERQ-CR* Emotion Regulation Questionnaire Cognitive Reappraisal, *ERQ-ES* Emotion Regulation Questionnaire Expression Suppression, *PSQI* Pittsburgh Sleep Quality Index.

**Figure 1:**
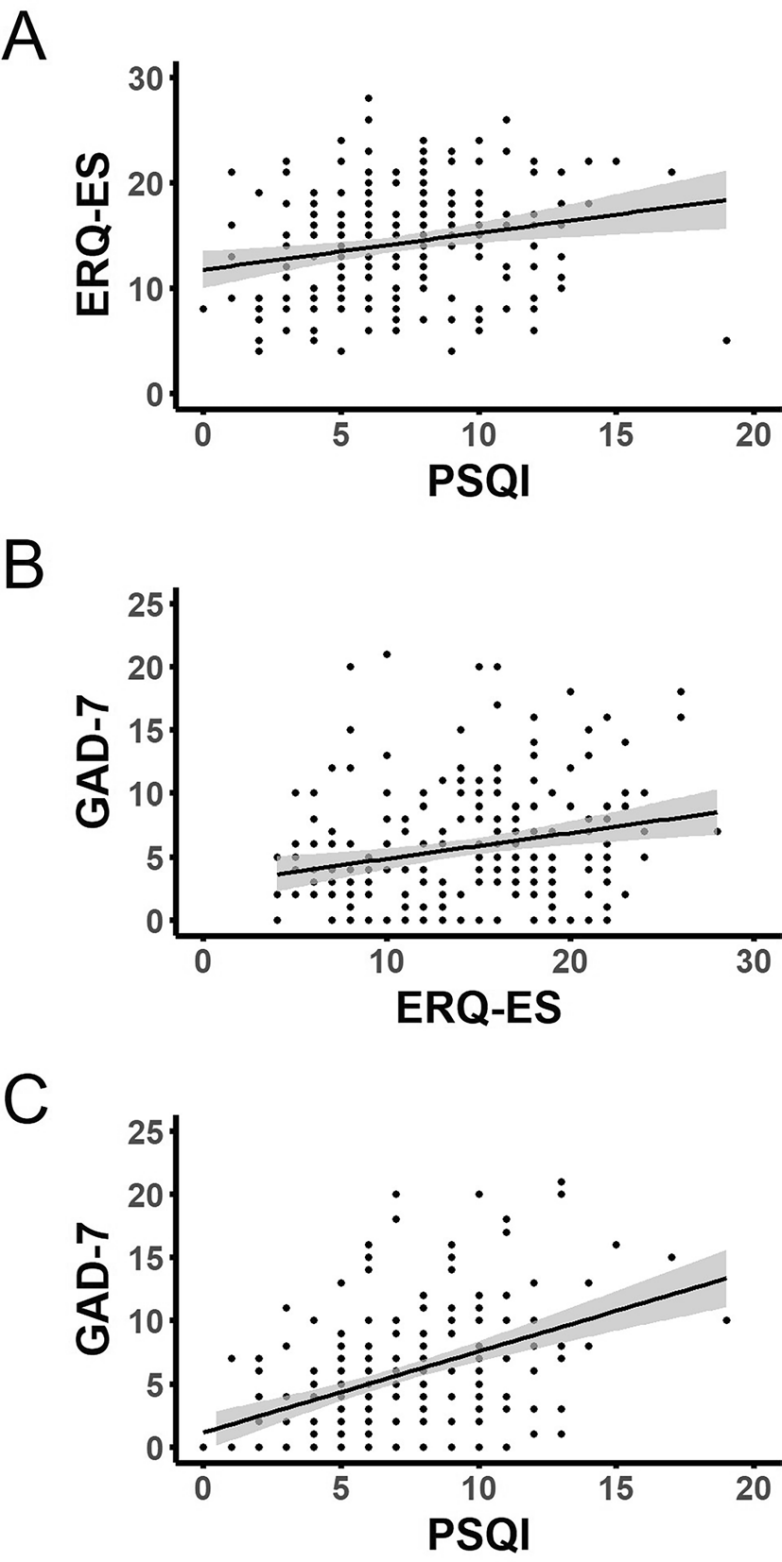
Scatter plots illustrating the relationships between sleep quality, expressive suppression and anxiety. Significant correlations were evident between sleep quality (PSQI) and expressive suppression (ERQ-ES; (**a**); expressive suppression and anxiety symptomology (GAD-7; **b**); and between sleep quality and anxiety symptomology (**c**). Trendlines show linear best fit. **p < 0.005, ***p < 0.0005, ****p < 0.0001 with Spearman’s Rank Correlation.

Pittsburgh Sleep Quality Index (PSQI) scores were positively correlated with Generalised Anxiety Disorder Questionnaire (GAD-7) scores, r_s_(203) = 0.424, *p* < 0.0001; Fig. 1, and Emotion Regulation Questionnaire Expressive Suppression (ERQ-ES) scores, r_s_(203) = 0.232, *p* = 0.0004; Fig. 1. As the PSQI is an inverse scale, with higher values indicating poorer sleep quality, this suggests that as quality of sleep worsens both habitual use of expressive suppression and generalised anxiety symptomology increase. Moreover, ERQ-ES was also positively correlated with GAD-7, r_s_(203) = 0.225, *p* = 0.0006; Fig. 1, indicating that higher levels of habitual expressive suppression use are associated with increased generalised anxiety symptomology. Emotion Regulation Questionnaire Cognitive reappraisal (ERQ-CR) scores, however, were not significantly correlated with either GAD-7, r_s_(203) = − 0.012, *p* = 0.432, or PSQI, r_s_(203) = − 0.039, *p* = 0.291. There was no indication of a significant relationship between habitual use of expressive suppression and habitual use of cognitive reappraisal, r_s_(203) = 0.023, *p* = 0.374.

Given the significant correlations identified between sleep quality, generalised anxiety symptomology and expressive suppression, we next performed mediation analysis to test the hypothesis that expressive suppression mediates the effect of sleep quality on anxiety symptomology. All assumptions for these regression-based analyses were met. A Durbin-Watson score of 2.039 indicated no autocorrelatation in the residuals, while the histogram and P-P plots of standardised residuals suggested that the residuals were normally distributed. Scatter plots of the dependent variable against standardised residuals, and of the standardised residuals against standardised predicted values, suggested that the assumptions of linearity and homoscedasticity of residuals were met. No issues with multicolinearity were present; all correlation coefficients were < 0.6, the variance inflation factor was 1.046 and tolerance was 0.956.

**Figure 2:**
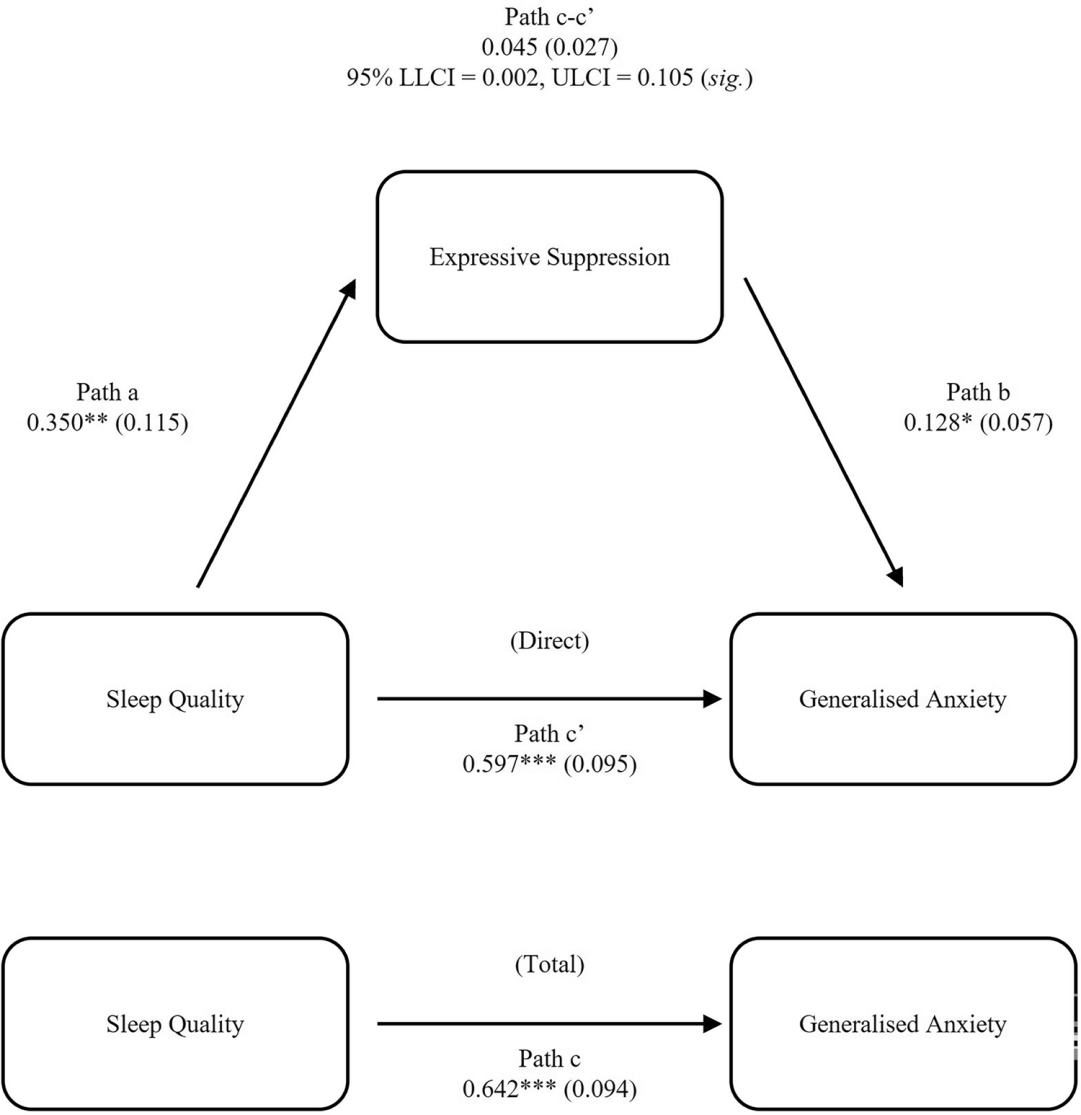
Expressive suppression partially mediates the effect of sleep quality on generalised anxiety. Data values are regression betas for specific pathways, with standard errors shown in parentheses. *LLCI* lower limit of 95% confidence interval; *ULCI* upper limit of 95% confidence interval. *p < 0.05, **p < 0.005, ***p < 0.0001. ‘*sig.*’ = indirect effect of sleep quality on generalised anxiety, mediated by expressive suppression, is significant, based on boot-strapping confidence intervals.

In a mediation model constructed with PSQI as the independent variable, ERQ-ES as the mediator and GAD-7 as the dependent variable (Fig. 2), sleep quality significantly predicted expressive suppression, β = 0.350, *SEM* = 0.115, *p* = 0.0026; Fig. 2, path a. Expressive suppression significantly predicted anxiety, β = 0.128, *SEM* = 0.057, *p* = 0.0271; Fig. 2, path b. The total effect of sleep quality on anxiety was also significant, β = 0.642, *SEM* = 0.094, *p* < 0.0001; Fig. 2, path c, demonstrating that regardless of the effect of expressive suppression on anxiety, sleep quality significantly predicted anxiety. When the effect of expressive suppression on anxiety was accounted for, the direct effect of sleep quality on anxiety remained significant, β = 0.597, *SEM* = 0.095, *p* < 0.0001; Fig. 2, path c’. A 10,000-sample bootstrap approach integrated within the model also revealed a significant indirect effect of sleep quality on anxiety, mediated by expressive suppression, β = 0.045, *SEM* = 0.027, 95% CI = 0.002, and 0.105. The model outcome, therefore, suggests partial mediation of the effect of sleep quality on anxiety by expressive suppression, with ~ 21% of the variance in anxiety accounted for by sleep quality and expressive suppression (*R*^*2*^ = 0.208).

## Discussion

The current study investigated whether two different ER strategies; expressive suppression and cognitive reappraisal, mediate the relationship between sleep quality and generalized anxiety symptomology within a non-clinical population. Worse sleep quality was correlated with increased use of expressive suppression and more severe anxiety symptomology. Increased use of expressive suppression was also correlated with more severe anxiety symptomology, whilst cognitive reappraisal was not correlated with sleep quality or anxiety symptoms. Novel regression-based mediation analysis demonstrates that expressive suppression, often theorized to be a maladaptive ER strategy, partially mediates the influence of sleep quality on anxiety symptomology. Compared to individuals with a clinical diagnosis of generalized anxiety disorder, our sample had reduced GAD-7 scores (5.7 vs 13.4)^40^, slightly reduced PSQI scores (7.1 vs 7.4^38^) and increased ERQ cognitive reappraisal scores (28.3 vs 20.8^40^ and 22.6^41^). To the authors knowledge, no study has reported expression suppression scores from the ERQ within a generalised anxiety disorder sample.

The finding that reduced sleep quality predicts increased anxiety symptomology is consistent with previous research^9–11,16–20^. Prior work investigating general ER difficulties, rather than the impact of *specific ER strategies*, has suggested that general ER difficulties mediated the relationship between GAD and some sleep-related outcomes, but not sleep quality^38^. Here, adopting regression-based mediation models, we extend these findings by demonstrating that habitual use of a specific ER strategy can mediate the relationship between sleep quality and anxiety symptomology. Specifically, we show that expressive suppression, but not cognitive reappraisal, partially mediates the influence of sleep quality on anxiety symptomology within a non-clinical adult population, extending prior work in an adolescent population. In addition to expressive suppression partially mediating the effect of sleep quality on anxiety symptomology, there is also a statistically significant direct effect of sleep on anxiety, even when this partial mediation is accounted for.

Related work has investigated how the interaction between ER and anxiety symptomology may be associated with insomnia symptom severity. Kirwan et al.^42^ demonstrated that as anxiety symptomology increased so did insomnia symptoms in those with high ER difficulties. However, for people with low levels of ER difficulty, changes in anxiety symptomology were not associated with an increase in insomnia symptoms. This finding therefore suggests that maladaptive ER may be required for anxiety symptoms to have a detrimental impact on insomnia-related symptomology. Consequently, this indicates it may be important to simultaneously target anxiety-related symptoms and ER difficulties especially in people with insomnia. However, specific ER strategies were not investigated, and this is an avenue for future research.

Previous research has predominantly suggested that increased use of adaptive ER strategies including cognitive reappraisal are associated with decreased anxiety symptomology^21,22,25–27,29^. The current study suggests that, within our sample, cognitive reappraisal is not associated with anxiety symptomology. Recent work has also shown that cognitive reappraisal is not associated with anxiety symptomology^43^, and meta-analytic research has shown that the influences of adaptive ER strategies (e.g., cognitive reappraisal) on anxiety symptomology are weaker than the impact of maladaptive ER strategies including expressive suppression^21^. In the current study, cognitive reappraisal was also not associated with sleep quality. Previous research adopting self-report questionnaires also suggests that cognitive reappraisal is not associated with sleep quality^28,30^, although experimentally determined cognitive appraisal ability has been shown to be associated with sleep quality^31^. The above findings therefore suggest it is possible that specific ER strategies (e.g., expressive suppression) are more strongly associated with sleep quality and anxiety symptomology than other strategies (e.g., cognitive reappraisal). Consequently, research that identifies those ER strategies most associated with the aetiology of sleep and anxiety disorders may help to develop more effective treatments and interventions. The current study suggests that targeting increased use of expressive suppression rather than cognitive reappraisal may be a more effective strategy in reducing anxiety symptoms in a non-clinical sample. A recent study compared the effectiveness of internet cognitive behavioural therapy (iCBT) for insomnia against iCBT for anxiety in people with comorbid insomnia and anxiety^44^. At the end of treatment, whilst both therapies were equally successful in reducing anxiety symptomology, greater reductions in insomnia symptoms were evident in the insomnia treatment condition. This finding suggests that for people with comorbid anxiety and insomnia, iCBT treatments aimed at improving insomnia may be more effective than treatments targeting anxiety symptomology. In a similar manner, the partial mediation evident in the current work suggests that whilst targeting both expressive suppression and sleep quality may be optimal for reducing anxiety symptomology, targeting improvements in sleep quality may have a greater impact on anxiety symptomology than targeting reduced use of expressive suppression. It should also be noted that associations between sleep quality/disturbances and psychological outcomes are not solely observed in anxiety and anxiety disorders. For instance, such associations are evident in people with hoarding symptoms^45^ and psychotic symptoms^46^. Future research is needed to investigate if simultaneously targeting ER strategy use and insomnia may result in more efficacious treatments and interventions.

The current work furthers our understanding regarding the associations between sleep quality, ER strategy use and anxiety symptomology in a non-clinical sample providing the basis for further research in clinical populations with sleep disorders and/or affective disorders including GAD. However, a limitation is the use of a cross-sectional design. Future work adopting experimental, longitudinal and intervention designs are required to provide causal inferences. Further limitations include the use of an opportunity sample and the exclusion of people with psychiatric illness. Moreover, whilst the current study is the first to test if specific ER strategies can mediate the influence of sleep quality on anxiety symptomology, it did not include the full range of ER strategies. Additional ER strategies have been associated with sleep quality^47^ and anxiety^48^ and the inclusion of additional strategies are needed in future investigations. Moreover, given that it has been argued flexible (adaptive) use of ER strategies is central to wellbeing^49^, and that successful ER is characterised by flexible adjustment of ER strategies^50^, ER research should also assess inter- and intra-individual variability in the flexible use of ER strategies, i.e., moving beyond investigations focussing on the habitual use of individual strategies and instead investigating the deployment of shifting ER strategies across contextual and situational demands.

Adopting mediation analyses, we show that a specific ER strategy, expressive suppression, partially mediates the impact of sleep quality on anxiety symptomology. This finding suggests that targeting improvements in the use of specific ER strategies, especially in those with dysregulated ER, along with simultaneously improving sleep quality, may help to develop more efficacious interventions and treatments aimed at reducing anxiety symptomology.

## Methods

### Participants

Using MedPower^51^, a sample size calculation for a mediation model assuming statistical power of 0.80 with an alpha criterion of 0.05 and an effect size of 0.25 suggested that a minimum sample of 156 participants was required. Adopting an opportunity sample, 203 participants (140 female, 62 males, 1 not disclosed) aged 18–73 years (*M* = 32.6, *SD* = 14.85) participated in the study. Participants were recruited via online adverts placed on social media platforms. People with a diagnosed psychiatric condition were excluded. This exclusion criteria was detailed in our Participant Information Sheet and participants were required to confirm if they had been diagnosed with a psychiatric condition within our online survey. If a participant had received a psychiatric condition diagnosis, including for a sleep disorder (e.g., insomnia), they were ineligible to participate in the study and unable to complete the survey. Participants read a Participant Information Sheet before providing informed consent. Ethical approval was obtained from the School of Health Sciences and School of Health and Society Ethics Committee at University of Salford, UK (HST2021-040). The study was performed in accordance with relevant guidelines and regulations.

### Design

The study adopted a cross-sectional correlational design consisting of four variables: sleep quality, cognitive reappraisal, expressive suppression, and generalised anxiety. The study was not preregistered.

### Materials

Participants completed the ERQ^24^ to assess habitual use of cognitive reappraisal and expressive suppression, the PSQI^52^ to assess sleep quality, and the GAD-7^53^ to record a measure of generalized anxiety symptomology. The GAD-7 is a 7-item questionnaire that measures respondents’ severity of generalized anxiety. Respondents are required to rate the extent to which a statement describes how they feel on a 4-point Likert scale ranging from 0 (not at all sure) to 3 (nearly every day). An example of a statement from this questionnaire is “Feeling nervous, anxious or on edge”. The minimum possible score is 0 and indicates lower levels of anxiety. The maximum possible score is 21 and indicates higher levels of anxiety. A score is calculated by summing all responses. The GAD-7 demonstrates good internal consistency for clinical samples (α = 0.92) and the general population (α = 0.89) as well as test–retest reliability (α = 0.83)^53,54^. Within the current sample the GAD-7 demonstrated a reliability score of α = 0.88.

The ERQ assesses the tendency of respondents to regulate their emotions using two different strategies, expressive suppression and cognitive reappraisal. The questionnaire uses 7-point Likert scales (1—strongly disagree to 7—strongly agree), presenting 10-items as two subscales to independently measure habitual use of cognitive reappraisal (6 items: e.g., “I control my emotions by changing the way I think about the situation I’m in”) and expression suppression (4 items: e.g., “I keep my emotions to myself”). This yields minimum and maximum possible scores for cognitive reappraisal of 6 and 42, representing lower and higher habitual use of cognitive reappraisal, respectively, and expressive suppression minimum and maximum possible scores of 4 (lower use of expressive suppression) and 28 (higher use of expressive suppression). ERQ exhibits reliability of α = 0.70 for cognitive reappraisal and α = 0.73 for expressive suppression, and a test–retest reliability of α = 0.69 for both subscales. Within the current sample the ERQ demonstrated reliability score of α = 0.84 for the cognitive reappraisal subscale and a reliability score of α = 0.74 for the expressive suppression subscale.

The PSQI is used to assess sleep quality using 18-items that contribute to 7 different components. Four are presented as direct questions, requiring either the time or duration of parameters related to sleep (e.g., “During the past month, how long (in minutes) has it usually taken you to fall asleep each night?”), and 14 are provided as 4-point Likert scales (e.g., “During the past month, how often have you had trouble sleeping because you cannot breathe comfortably”). Component scores are derived directly from individual Likert scales or via calculation from multiple individual-item Likert scales and/or from time/duration responses. The 7 component scores can range from 0 (no difficulty with that sleep parameter) to 3 (severe difficulty with that parameter) and are ultimately summed to provide a global PSQI score that can range from 0 (higher quality sleep) to 21 (lower quality sleep). Participants are required to respond based on “usual sleep habits during the past month only” and to “indicate the most accurate reply for the majority of days and nights in the past month”. PSQI exhibits a high level of internal homogeneity (α = 0.83) and diagnostic sensitivity to distinguish good vs poor sleepers of 89.6% and 86.5%, respectively (*k*—0.75, *p* < 0.001). PSQI also includes an additional item, consisting of 5 sub-items that does not contribute a component or the global PSQI score, but records the presence of a bed partner or roommate and potential disruption related to this. Within the current sample the PSQI demonstrated a reliability score of α = 0.70.

### Procedure

The questionnaires were uploaded onto Online Surveys (JISC), an online system used to collect survey data. Before completing the questionnaires, participants viewed a Participant Information Sheet and provided informed consent. All participants had the opportunity to seek clarification from the research team before completing the questionnaires.

### Statistical analysis

All statistical analyses were conducted using SPSS (version 26). Data were assessed to check the assumptions required for parametric analyses and then initially investigated using Spearman’s Rank-Order Correlation (one-tailed). Parameters with statistically significant relationships were subsequently included in regression-based mediation models, with appropriate assessments of the assumptions required for multiple regression. ERQ-CR subscale scores were not significantly correlated with any other variables so was not included in any further analyses (see “Results”). The PROCESS v3^55^ extension to SPSS was used to construct and evaluate mediation models, with PSQI global score as the independent variable (X), GAD-7 score as the dependent variable (Y) and ERQ-ES subscale score as the mediator (M). This approach allowed us to investigate both whether sleep quality and expressive suppression predict anxiety, and whether habitual use of expressive suppression significantly mediates the effect of sleep quality on anxiety.

## Data Availability

The datasets generated during and/or analysed during the current study are available in the Open Science Framework repository (https://osf.io/nkfgz/).
